# Peroral endoscopy during the COVID‐19 pandemic: Efficacy of the acrylic box (Endo‐Splash Protective (ESP) box) for preventing droplet transmission

**DOI:** 10.1002/jgh3.12438

**Published:** 2020-10-21

**Authors:** Kuniyo Gomi, Masatsugu Nagahama, Erika Yoshida, Yuichi Takano, Yuichiro Kuroki, Yorimasa Yamamoto

**Affiliations:** ^1^ Division of Gastroenterology Showa University Fujigaoka Hospital Yokohama Japan

**Keywords:** COVID‐19, droplet transmission, endoscopy: Upper gastrointestinal

## Abstract

**Background and Aim:**

Appropriate personal protective equipment should be worn in all clinical settings during the COVID‐19 pandemic because anyone could be carrying SARS‐CoV‐2. Peroral endoscopy is the procedure potentially generating large volumes of aerosols through stimulation of patient coughing. The present study investigated the efficacy of a specially designed acrylic box (Endo‐Splash Protective [ESP] box) for preventing droplet transmission as part of droplet precautions for endoscopists and assistants during routine endoscopy for potential asymptomatic carriers or emergent endoscopy for confirmed or suspected COVID‐19 patients.

**Methods and Results:**

ESP box was fabricated for use in peroral endoscopy performed with the patient in either a left lateral or prone position. Circular ports were created, one for scope insertion and one for each of the assistant's hands. Simulated droplets were released inside ESP box, and the number of droplets was counted every 100/3 ms for 5 s pre‐release and post‐release at the positions of the endoscopist and assistant. The experiment was repeated eight times at the endoscopist and assistant positions, and the median numbers of droplets were calculated and compared. No significant differences were observed between the median number of droplets counted for 5 s prerelease and postrelease at either the endoscopist (*P* = 0.239) or assistant (*P* = 0.576) positions. We could block the droplets by using ESP box.

**Conclusions:**

The present findings suggest that use of ESP box during peroral endoscopy may reduce endoscopist and assistant droplet exposure, potentially reducing the risk of droplet transmission to healthcare professionals performing peroral endoscopy during the COVID‐19 pandemic.

## Graphical abstract

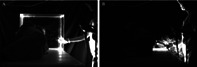

## Introduction

Coronavirus disease 2019 (COVID‐19), caused by severe acute respiratory syndrome coronavirus 2 (SARS‐CoV‐2), a novel coronavirus, has spread globally since the initial outbreak in Wuhan City, Hubei Province, China, in December 2019. The World Health Organization declared COVID‐19 a Public Health Emergency of International Concern on 30 January 2020 and subsequently labeled the outbreak a pandemic on 11 March. In Japan, the first patient was reported on 16 January, COVID‐19 was classified as a Designated Infectious Disease on 1 February, and a state of emergency was declared on 7 April.

The causative agent of COVID‐19, SARS‐CoV‐2, mainly causes respiratory illness, but it is thought to be less pathogenic than Middle East respiratory syndrome (MERS) or severe acute respiratory syndrome (SARS). The incubation period for COVID‐19 ranges from 1 to 14 days, with symptom onset around 5 days after exposure. Unlike SARS and MERS, COVID‐19 is highly infectious after onset, causing community transmission.^1^ The infectious period is thought to last from 2 days before until 7 to 14 days after symptom onset. SARS‐CoV‐2 virus replication occurs in the upper and lower respiratory tract, and severe cases are associated with a high viral load and long shedding period, with virus genes frequently detected 3 to 4 weeks after onset.^2^ The primary route of infection is droplet transmission, and infection can occur in poorly ventilated environments even in the absence of coughing and sneezing. Whereas symptomatic individuals are the primary agents for transmitting infection, asymptomatic carriers also pose an infection risk. Airborne transmission is unlikely; however, there are indications of wide‐ranging infection beyond the conventional concept of droplet transmission.^3^

Therefore, because anyone could be carrying SARS‐CoV‐2, appropriate personal protective equipment (PPE) should be worn in all clinical settings during the COVID‐19 pandemic.^4^ Furthermore, droplet and contact precautions should be implemented in addition to standard precautions when examining confirmed or suspected COVID‐19 patients. Though caps, gowns, gloves, and PPE that cover the eyes, nose, and mouth are routinely worn, N95 respirators should be used instead of surgical masks in situations where large volumes of aerosols are likely to be temporarily generated.^5^

According to the Japanese Society for Infection Prevention and Control guidance for exposure risk assessment and countermeasures for healthcare providers (HCPs),^5^ HCPs who are in close contact with a confirmed COVID‐19 patient without a mask for 15 min or longer, perform a procedure that generates a large volume of aerosol even while wearing a surgical mask, or are present in the treatment room during such a procedure are at moderate risk of infection and should be excluded from work for 14 days. One such aerosol‐generating procedure is gastroscopy, particularly peroral gastroscopy, which can stimulate patient coughing. An HCP who does not use an N95 respirator when performing peroral or transnasal endoscopy on a COVID‐19 patient is considered to be at moderate risk.

The Gastrointestinal Endoscopy in the Era of the Acute Pandemic of COVID‐19: Recommendations by the Japan Gastroenterological Endoscopy Society^6^ state that endoscopy should only be performed on confirmed or suspected COVID‐19 patients if urgently required. Indications for unavoidable emergent endoscopy include gastrointestinal bleeding with shock requiring endoscopic hemostasis and biliary tract infection requiring endoscopic biliary drainage. Furthermore, during the pandemic, the indications for endoscopy must be carefully examined even in cases where COVID‐19 is not clinically suspected, and endoscopy postponement or cancellation should be considered in non‐emergent cases. It is also possible that some patients undergoing preoperative or other non‐emergent but unavoidable endoscopy may be asymptomatic carriers. With the additional burden of PPE shortages and requirements for post‐endoscopic disinfection of the treatment room, HCPs require simpler, more reliable infection precautions. Kagawa *et al*.^7^ reported for the first time a plastic cube for endoscopes that made it possible to reduce aerosol exposure to operators. We created an acrylic box with holes for the endoscope and the assistant's hands.

The present study investigated the efficacy of a specially designed acrylic box (Endo‐Splash Protective box [ESP box]) for the prevention of droplet transmission as part of droplet precautions for operators and assistants during routine endoscopy for potential asymptomatic carriers or emergent endoscopy for confirmed or suspected COVID‐19 patients.

## Methods

**Figure 1:**
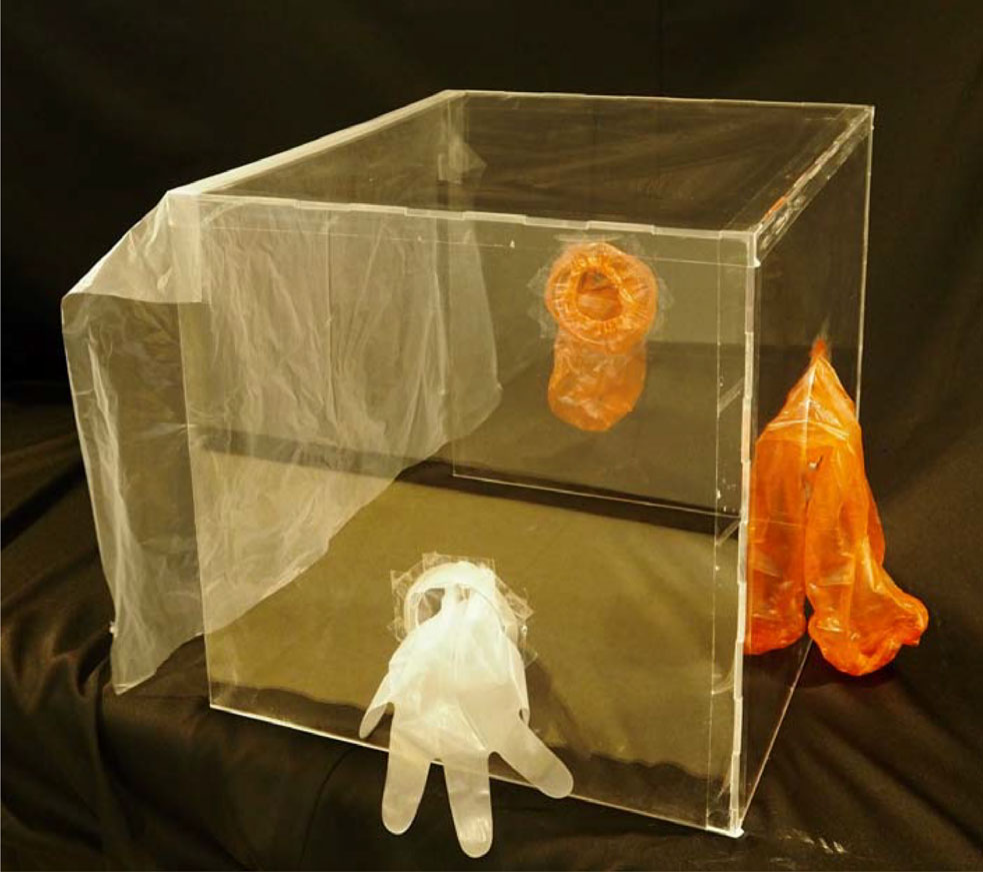
Photograph of Endo‐Splash Protective box.

An acrylic box measuring 500 mm (height) × 500 mm (width) × 600 mm (depth) was fabricated from 5‐mm acrylic sheet for use in peroral endoscopy performed with the patient in either a left lateral or prone position (COMNET Corporation, Hyogo, Japan). Circular ports were created, one for scope insertion on one side wall (diameter, 80 mm) and one for each of the assistant's hands on the opposite side wall and end wall nearest the top of the patient's head (diameter, 110 mm). We made the hole for inserting the endoscope into a circular shape in order to improve the operability of the endoscope. A latex glove was taped to the scope insertion port, and a hole was opened in the tip of the middle finger of the glove through which the scope could be inserted into the box. Vinyl arm covers were taped to the two ports for the assistant's hands to access the box. A vinyl curtain was attached to the open side of the box positioned over the patient's shoulders (Fig. 1).

**Figure 2:**
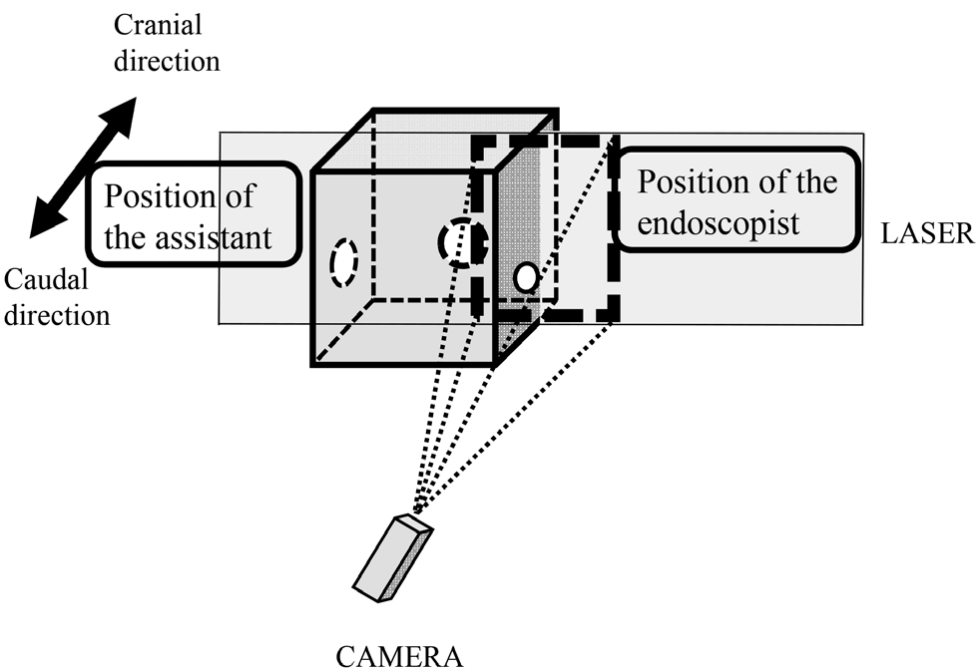
Method of counting droplets.

To replicate droplet dispersion inside the acrylic box, an adjustable air blower was used to simulate a cough of typical velocity (10 m/s)^8^ with 0.1 g titanium dioxide powder (particle diameter, 0.254 μm) as a tracer. The number of tracer particles (simulated droplets) was counted for 5 s pre‐release (before releasing the tracer particles) and post‐release (after releasing the tracer particles) at the positions of the endoscopist and assistant outside the acrylic box. The experiment was repeated eight times at the endoscopist and assistant positions, and the median numbers of simulated droplets were calculated and compared. The droplets were counted by the leather light scattering method, which has been used in previous studies.^9^ The number of simulated droplets for 5 s pre‐release and post‐release was counted and analyzed every 100/3 ms with Laser Light Scattering. We used a light source (2 W output [Continuous Wave] PIV Laser G2000; Katokoken Co., Ltd., Kanagawa, Japan) placed at the endoscopist and assistant positions, a camera (ParticleViewer 2‐II; Katokoken Co., Ltd.), and image analysis software (KM2.0; Katokoken Co., Ltd.; Fig. 2).

Peroral upper gastroscopy was also performed on healthy volunteers with 1 g of corn starch (particle diameter, 3–35 μm) placed in front of the mouth, and photos and video images were recorded to visualize and compare simulated droplet dispersion with and without the acrylic box. Images were recorded using a light source (2 W output [Continuous Wave] PIV Laser G2000; Katokoken Co., Ltd.) and camera (ParticleViewer 2‐II; Katokoken Co., Ltd).

### *Statistical analyses*

The Wilcoxon signed‐rank test was used for continuous parameters with a non‐normal distribution. *P*‐values <0.05 were considered significant in two‐sided tests. SPSS (IBM, New York, NY, USA) for Windows was used for the statistical processing.

## Results

**Figure 3:**
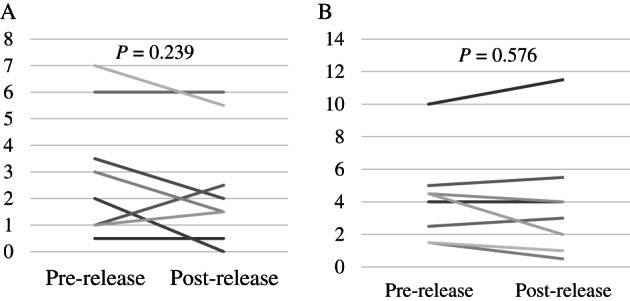
(a) The number of tracer particles of the position of the endoscopist (b) the number of tracer particles of the position of the assistant.

No significant differences were observed between the median number of simulated droplets counted every 100/3 ms for 5 s pre‐release and post‐release at either the endoscopist (*P* = 0.239; Fig. 3a) or assistant (*P* = 0.576; Fig. 3b) positions. In the absence of the ESP box, there were droplets in the endoscopist's position, and the median numbers of simulated droplets increased from 0 (range, 0–7) pre‐release to 48.5 (4–2592) post‐release.

The latex glove was taped to the scope insertion port, and a hole was opened in the tip of the middle finger of the glove through which the scope could be inserted into the box. However, in some experiments, part of the tape holding the glove to the box became detached, creating a small gap between the glove and the acrylic box. On such occasions, the median numbers of simulated droplets increased from 4 (range, 0–9) pre‐release to 10.5 (1–25) post‐release.

**Figure 4:**
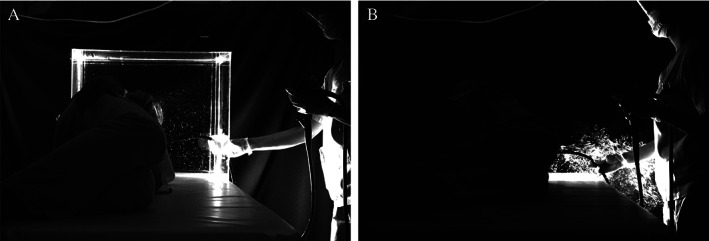
Static images to visualize simulated droplet dispersion during peroral endoscopy (a) with Endo‐Splash Protective (ESP) box (b) without ESP box.

Static images to visualize simulated droplet dispersion during peroral endoscopy performed with and without ESP box demonstrated that droplet exposure occurred without (Fig. 4a), but not with ESP box (Fig. 4b) at the position of the endoscopist (Supplementary video).

## Discussion

The primary route of infection of SARS‐CoV‐2, the causative agent of COVID‐19, is droplet transmission, but contact transmission is also possible. Though symptomatic individuals are the primary agent for transmitting infection, asymptomatic carriers also pose an infection risk.^4^ The possibility that anyone could be carrying SARS‐CoV‐2 must be taken into account during medical examinations conducted during the COVID‐19 pandemic. Peroral and transnasal endoscopy potentially generate large volumes of aerosols through stimulation of patient coughing. Therefore, the indications for endoscopy must be carefully examined during the pandemic, even in cases where COVID‐19 is not clinically suspected, and the possibility that some patients undergoing unavoidable endoscopy may be asymptomatic carriers must be considered. Droplet precautions are important to reduce the risk of infection for healthcare professionals performing upper gastroscopy during the pandemic.

With droplet transmission, infection occurs via direct inhalation of droplets released by patients within a very close range. Comprising aqueous particles, these respiratory droplets are larger and fall much more rapidly than droplet nuclei, remaining in the air for only a short period of time. Droplets released into the air fall onto surfaces and objects within 1 to 2 m of the source. Therefore, for prevention of droplet transmission, precautions for shared air close to the patient are more important than those for the entire room.^10^ When droplets land on the oral, nasal, and ocular mucosa, the pathogenic microbes within begin to proliferate, causing symptom onset. Therefore, unlike airborne transmission (droplet nuclei transmission), precautions are required against the danger of direct exposure to patient fluids, including further protection for the face, particularly the eyes, in addition to masks. Droplet transmission can be blocked by covering the source and by physically preventing released droplets from reaching susceptible individuals. The former is highly effective and the least expensive approach and, thus, should be the primary measure implemented by encouraging infected patients to wear a surgical mask.^11^ However, the latter approach has to be adopted for peroral and transnasal gastroscopy, during which the patient cannot wear a surgical mask. In the present study, ESP box was devised to cover patients undergoing gastroscopy as a method of physically preventing released droplets from reaching the HCPs.

No significant differences were observed between the number of simulated droplets counted pre‐ and post‐release at either the endoscopist or assistant positions outside ESP box. Particles were visible in the post‐release images; however, dust and other particles were also present within the imaging range before release of the simulated particles; thus, it was possible to demonstrate the preventive efficacy of ESP box by comparing the number of simulated particles counted pre‐ and post‐release. Since this verification was done in an environment with dust and other particles, it was impossible to reduce the number of particles to zero before release of the simulated particles, and the only way to do so was to compare pre‐ and post‐release the droplets. Visual observation also suggested that HCPs exposure to droplets during endoscopy was prevented.

**Figure 5:**
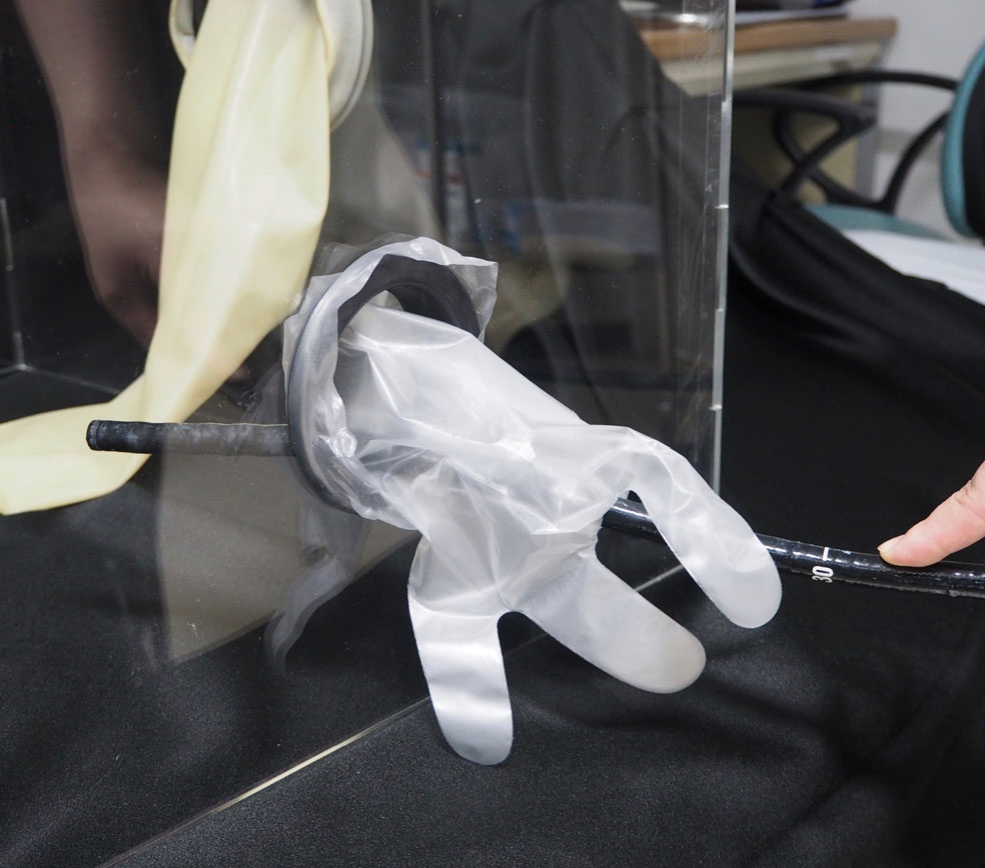
Photograph of Endo‐Splash Protective box (modified version).

In experiments when a small gap opened up between the latex glove and the scope insertion port, the number of simulated droplets clearly increased after release, suggesting that droplet exposure can occur with even small gaps. Based on these findings, the latex glove was attached to ESP box using a commercial drainage channel gasket through which the latex glove was tucked into the scope insertion port, rather than using tape to prevent gaps between the box and the glove (Fig. 5). Furthermore, the vinyl arm covers that were also attached with tape to the ports for the assistant's hands to access the inside of ESP box were replaced with glovebox gloves for use in experiments and fixed to the box through commercial drainage channel gaskets to prevent both gaps between the box and the gloves and exposure of the assistant's hands inside the box. The diameter of the ports for the assistant's hands was increased to 120 mm based on the size of the commercial drainage channel gaskets. The latex gloves and glove box gloves are disposable and the ESP box is wiped down with a chlorine sanitizer for reuse. The use of modified ESP box to perform endoscopy may reduce the risk of droplet infection to HCPs. During the COVID‐19 pandemic, ESP box may be applicable not only to screening endoscopy, but also to other endoscopies such as emergency endoscopic hemostasis, ERCP, EUS, and ESD. We are currently verifying the safety of the ESP box by obtaining consent for patients to undergo screening endoscopy. Once proven safe, we plan to use the ESP box for endoscopic procedures and to test the operability and safety.

In conclusion, the present findings suggested that use of ESP box during peroral endoscopy may reduce endoscopist and assistant droplet exposure, reducing the risk of droplet transmission to HCPs performing peroral endoscopy during the COVID‐19 pandemic.

## Supporting information


**Video S1.** Video to visualize the simulated droplet dispersion during peroral endoscopy.
